# Fracture fixation in extremity trauma with carbon fiber-reinforced polyetheretherketone (CFR-PEEK) plates: evidence today

**DOI:** 10.1007/s00068-021-01778-x

**Published:** 2021-09-06

**Authors:** George D. Chloros, Apostolos D. Prodromidis, Jo Wilson, Peter V. Giannoudis

**Affiliations:** 1 Academic Department of Trauma and Orthopaedic Surgery, School of Medicine, University of Leeds, Leeds, UK; 2 Invibio Biomaterial Solutions Ltd, Hillhouse International, Thornton-Cleveleys, Lancashire UK; 3 NIHR Leeds Biomedical Research Center, Chapel Allerton Hospital, Leeds, UK

**Keywords:** Fracture fixation, Carbon fiber plates, Polyetheretherketone, Outcomes, Complications

## Abstract

**Purpose:**

To compare the CFR-PEEK plates with conventional plates in fracture fixation with regards to clinical and radiological outcomes and complications.

**Methods:**

A systematic literature search was conducted in four online databases independently by two reviewers using the Cochrane methodology for systematic reviews. The identified relevant studies were assessed against predetermined inclusion/exclusion criteria. Independent data extraction and assessment of risk of bias and study quality was carried out.

**Results:**

Nine studies (patient *n* = 361) out of 6594 records were included for analysis: 2 RCTs (*n* = 63), 3 prospective cohort studies (*n* = 151), and 4 retrospective cohort studies (*n* = 147). Studies were grouped per anatomic area of fixation. Four studies (*n* = 200) examined fixation of proximal humerus fractures. Two studies (*n* = 74) examined fixation of distal radius fractures. Two studies (*n* = 53) assessed outcomes of fixation of distal femur fractures. One study (*n* = 87) assessed the outcomes of fixation of ankle fractures. All nine studies reported very high union rates (from 91% in distal femur to 100% in upper limb) for the CFR-PEEK plate groups and low complication rates. There was no significant difference in clinical outcomes, and rate of complications as compared to the conventional plate groups.

**Conclusion:**

CFR-PEEK plates have high union rates in extremity fracture fixation similar to conventional plates with comparable good clinical outcomes and a very low and comparable rate of complications. Considering their advantages, CFR-PEEK plates seem to be valid alternative to conventional plating.

**Supplementary Information:**

The online version contains supplementary material available at 10.1007/s00068-021-01778-x.

## Introduction

Carbon fibers are fibers of about 5–10 µm the majority of which is composed of carbon atoms. They have unique advantage properties, such as high stiffness, tensile strength, temperature tolerance, chemical resistance, and are lightweight [1, 2]. The widespread use of carbon fiber composites in many fields including aerospace, military, civil engineering, and sporting industries paved the way to expand its use in medicine [2], with numerous applications particularly in orthopaedics including spine, joint arthroplasty and orthopedic trauma [2, 3]. The use of carbon fiber-reinforced polyetheretherketone (CFR-PEEK) implants is an emerging field in orthopaedic surgery because of the numerous advantages this material offers compared to its conventional counterparts (e.g., stainless steel) and these can be summarized as follows: (1) modulus of elasticity close to bone, therefore avoiding stress-shielding and resultant bone resorption (2) radiolucency and therefore enhanced ability to accurately achieve fracture reduction and monitor healing, (3) decreased artefact in Magnetic Resonance Imaging Scans, (4) no metal allergy, and increased osteoinductive properties and biocompatibility with minimal implant-related inflammatory response, (5) absence of cold welding at the plate-screw interface, (4–11). Main disadvantages of these plates include: (1) They cannot be contoured intraoperatively (form memory property) [4, 5]; (2) the increased cost of production, although the commercial price is similar to the conventional metal implants [6]; and (3) radiolucency at the same time may compromise appropriate plate visualization which is crucial to assess position or hardware failure, however radiopaque tantalum markers have been developed as a remedy [7, 8].

However, despite those potential advantages and preliminary reports already dating back to the 1980s [9], and their use steadily increasing recently, limited amount of studies exist in the literature. There have been sporadic reviews on the subject [2, 3, 10], mostly reporting their general orthopedic applications throughout the body using a variety of implants but robust systematic reviews are missing. The purpose of this study therefore was to conduct a focused systematic review to report the outcomes and complications of CFR-PEEK plates used for the fixation of extremity fractures.

## Methods

For this systematic review, the Cochrane methodology for systematic reviews was followed [11]. The work was conducted with reference to a predefined protocol, which was registered with the PROSPERO database (https://www.crd.york.ac.uk/prospero/) (CRD42021245114). The strategy for the systematic literature search included: (i) searching of electronic bibliographic databases, and (ii) scrutiny of references of included studies and any identified systematic review. The following electronic bibliographic databases were searched on March 2021 with no publication year limit: MEDLINE—Interface: EBSCOhost; EMBASE—Interface: Ovidsp; CINAHL (1961 to present)—Interface: EBSCOhost; CENTRAL (1988 to present)—Interface: Cochrane Library. There was a language limit because of limited access to translators and resources. Therefore, only studies available in English language were included. Age was not set as a limit to the search because of the difficulty of setting specific search terms, but all titles and abstracts about children (age < 16 years) were excluded whilst screening. The search in all databases was performed with a combination of keywords in multiple searches. Keywords were combined with the Boolean operators OR and AND. The selected keywords and the strategy for combining these keywords in five searches are summarized in Table 1.

**Table 1:** Summary of strategy for search performed in all databases

Search	Set of keywords		Set of keywords
S1	Carbon fiber OR Carbon fibre	AND	Implant* OR material* OR biomaterial* OR polymer* OR composite*
S2	Carbon fiber-reinforced OR Carbon fibre-reinforced	AND	Implant* OR material* OR biomaterial* OR polymer* OR composite*
S3	PEEK	AND	Implant* OR material* OR biomaterial* OR polymer* OR composite*
S4	Carbon fiber OR Carbon fibre	AND	Orthop*
S5	PEEK	AND	Orthop*

The asterisk is a wildcard and is included in the search

### Inclusion/exclusion criteria

- *Study designs:* Any comparative study design was eligible. This included randomised controlled studies, prospective cohort studies, case control studies, and retrospective cohort studies. Excluded study designs included case reports, reviews, editorials, commentaries, personal opinions, surveys and retrospective case series.
- *Population*: The population included in the review were adults with an upper or lower extremity fracture who had surgical fixation with carbon fiber-reinforced plates.
- *Intervention/Comparators:* The intervention was surgical fixation of upper or lower extremity fractures with plate and screws and studies which compared outcomes of CFR plates with conventional plates were included.
- *Outcomes:* Outcomes included clinical outcomes (scores), radiographic outcomes, union (rates and/or time to union), and complications.

Based on the above inclusion/exclusion criteria, the titles of studies identified by the searches were screened for inclusion. Duplicate studies were removed. The abstracts of potential studies were then further screened and the full manuscripts of those studies still considered eligible were retrieved. The full text of studies, where a decision regarding inclusion could not be made from the title and abstract, were also retrieved. The reference lists of all selected articles (and of any other systematic review) were also examined for any additional articles not identified through the database search. Two reviewers assessed the search outputs independently. Any disagreements for inclusion were discussed between reviewers and if still unresolved with the senior experienced author.

### Data extraction and data analysis

Two reviewers extracted relevant data from the included studies using a standardised data extraction form and inputted onto an Excel spreadsheet. Where necessary, results were discussed with the senior author to decide for extraction. Extracted data included:

- Characteristics of studies: study design, level of evidence, year, country, setting, number of patients.
- Characteristics of included population: age, gender, body mass index (BMI), comorbidities.
- Side of fracture (left or right / dominant or non-dominant).
- Types of fractures and classification used.
- Outcomes examined and compared including clinical outcomes (scores), radiographic outcomes, range of motion (ROM), union (rates and/or time to union), and complications.
- Follow-up: duration and loss to follow-up.

Due to the inherent heterogeneity of the included studies and the different areas of fixation examined a meta-analysis could not be performed. A brief narrative analysis of the studies was performed, presenting study characteristics, populations, outcomes and measurements.

### Assessment of methodological quality of studies and quality of evidence

The methodological quality of each study was assessed as appropriate to the study design. For randomised controlled trials (RCTs), the Cochrane Risk of Bias Tool was applied [12]. For prospective comparative (cohort) studies, the Newcastle–Ottawa Scale (NOS) was used [13]. For retrospective cohort studies the revised and validated version of Methodological Index for Non-Randomised Studies (MINORS criteria) was applied [14]. Quality of evidence for the body of literature in the systematic review was assessed by two raters independently using the GRADE (Grading of Recommendations, Assessment, Development, and Evaluation) approach [15]. GRADE assesses the quality of evidence as high, moderate, low, or very low based on risk of bias, directness, consistency, precision, and reporting of bias [15]. Observational studies are considered low quality evidence but may be downgraded or upgraded according to GRADE recommendations.

## Results

### Findings of database searches

**Fig. 1:**
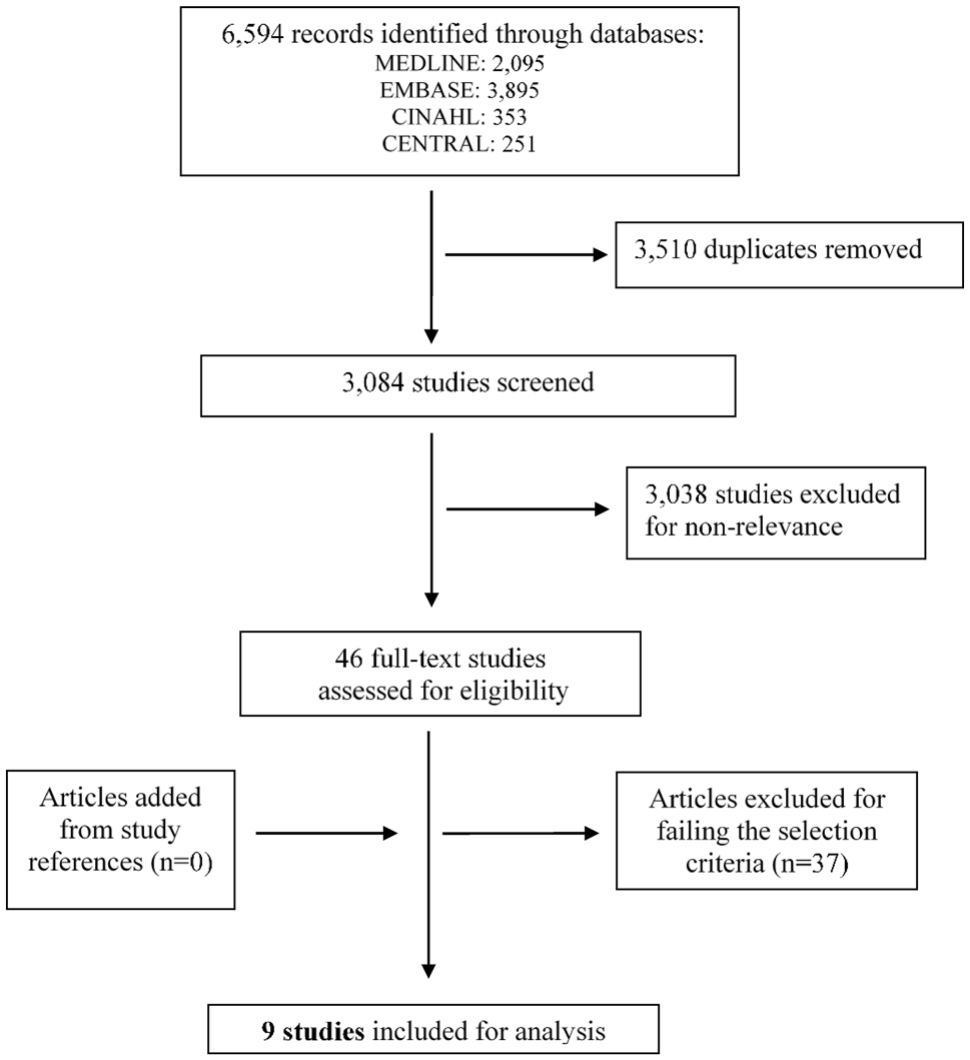
Methodology of identification and selection of studies (PRISMA flow chart) (14)

The searches identified 6594 records by title in total. For screening of the results an automated software was used (https://www.covidence.org/). After removal of 3,510 duplicates, 3,084 titles were screened. The screening process led to the initial selection of 46 studies based on information gathered from the titles and abstracts. A full-text review of these 46 articles and a thorough search of their references were performed. Finally, nine studies met the inclusion criteria and were used for analysis. Figure 1 shows the Preferred Reporting Items for Systematic reviews and meta-analyses (PRISMA) flow diagram used for identification of eligible studies [16].

### Characteristics of included studies

Table 2 summarizes the characteristics of the nine included studies, which were published between 2015 and 2020. The methodology of each study was classified according to Mathes and Pieper [17]. There were two randomised controlled trials (RCTs) (*n* = 63) [18, 19], and three prospective cohort studies (*n* = 151) [20–22]. The remaining four were retrospective cohort studies (*n* = 147) [23–26]. The total number of participants included in the analysis from these studies was 361. Comparison groups included a group of patients fixed with a carbon fibre-reinforced polyetheretherketone (CFR-PEEK) plate and a group of patients treated with a conventional stainless-steel plate. One study only had as a control group the contralateral uninjured extremity [21].

**Table 2:** Characteristics of all included studies in the systematic review

Lead author (year)	Study design Level evidence Country	Groups/implants/company	Sample size (*n*)	Gender	Age (years)	Side	Patient characteristics
Proximal humerus							
Ziegler (2019) [19]	RCT Level I Germany	Group 1 PEEK Power Humeral Fracture Plate (Arthrex, Naples, Florida, USA) Group 2 Proximal Humerus Internal Locking System—PHILOS (Depuy Synthes, West Chester, PA, USA)	63	Overall 13M: 50F Group 1 6M: 26F Group 2 7M: 24F	Group 1 Mean: 61.8 Range: 49.4–74.2 Group 2 Mean: 60.9 Range: 48.5–73.3	Overall 32D:31ND Group 1 15D:14ND Group 2 17D:17ND	NSD: gender, age, BMI, ASA
Padolino (2018) [25]	Retrospective cohort Level III Italy	Group 1 Diphos H CFR-PEEK plate (Lima Corporate, Italy) Group 2 Proximal Humerus Internal Locking System—PHILOS (DePuy Synthes, Umkirch, Germany)	42	Overall 16M: 26F Group 1 9M: 12F Group 2 7M: 14F	Group 1 Mean: 57.4 Range: 41.0–78.0 Group 2 Mean: 55.8 Range: 22.0–78.0	Overall 39D:3ND Group 1 19D:2ND Group 2 20D:1ND	NSD: gender, age, BMI
Katthagen (2017) [22]	Prospective cohort Level II Germany	Group 1 PEEK Power Humeral Fracture Plate (Arthrex, Naples, Florida, USA) Group 2 Proximal Humerus Internal Locking System—PHILOS (DePuy Synthes, Umkirch, Germany)	42	Overall 14M: 28F Group 1 7M: 14F Group 2 7M: 14F	Group 1 Mean: 66.8 Range: 56.9–76.7 Group 2 Mean: 67.4 Range: 57.7–77.1	Overall 25D:17ND Group 1 12D:9ND Group 2 13D:8ND	NSD: gender, age
Schliemann (2015) [26]	Retrospective cohort Level III Germany	Group 1 Diphos H CFR-PEEK plate (Lima Corporate, Italy) Group 2 Proximal Humerus Internal Locking System—PHILOS (DePuy Synthes, Umkirch, Germany)	53	NE	N/E	NR	NR
Distal radius							
Guzzini (2018) [21]	Prospective cohort Level II Italy	“Piccolo” Distal Radius Plate (CarboFix® Orthopaedics Ltd, Israel)	22	Overall 8M: 14F Group 1 8M: 14F Group 2 8M: 14F (contralateral)	Group 1 Mean: 50.8 Range Group 2 Mean: 50.8 SD: 10.34	NR	NSD: gender, age
Perugia (2017) [18]	RCT Level I Italy	Group 1 CarboFix CFR-PEEK distal radius volar locking plate (CarboFix® Orthopaedics Ltd, Israel) Group 2 Acu-Lock Volar Distal Radius Plate (Acumed Ltd., USA)	30	Overall 9M: 21F Group 1 5M: 10F Group 2 4M: 11F	Group 1 Mean: 56.8 Range: 32.0–71.0 Group 2 Mean: 52.6 Range: 35.0–64.0	Overall 10D:20ND Group 1 4D:11ND Group 2 6D:9ND	NSD: gender, age
Distal femur							
Byun (2020) [23]	Retrospective cohort Level III USA	Group 1 CarboFix CFR-PEEK distal femur locking plate (CarboFix® Orthopaedics Ltd, Israel) Group 2 VA-LCP Curved Condylar Plate (DePuy Synthes, Paoli, PA)	30	Overall 16M: 14F Group 1 6M: 3F Group 2 10M: 11F	Group 1 Mean: 49.8 Range: 23.0–80.0 Group 2 Mean: 54.9 Range: 18.0–89.0	NR	NSD: gender, age, BMI, smoking, diabetes
Mitchell (2018) [24]	Retrospective cohort Level III USA	Group 1 CarboFix CFR-PEEK distal femur locking plate (CarboFix® Orthopaedics Ltd, Israel) Group 2 VA-LCP Curved Condylar Plate (DePuy Synthes, Paoli, PA)	22	Overall 6M: 16F Group 1 3M: 8F Group 2 3M: 8F	Group 1 Mean: 71.7 Range: 51.0–89.0 Group 2 Mean: 57.3 Range: 27.0–86.0	NR	NSD: gender, smoking, PVD SSD: age, diabetes
Ankle							
Guzzini (2017) [20]	Prospective cohort Level II Italy	Group 1 CFR-PEEK ankle radiolucent plate (not stated) Group 2 Stainless steel ankle plate (not stated)	87	Overall 25M: 62F Group 1 14M: 32F Group 2 11M: 30F	Group 1 Mean: 56.8 Range: 54.46–59.14 Group 2 Mean: 58.3 Range: 59.14–61.85	NR	NSD (calculated): gender, age

*CFR-PEEK* carbon fibre-reinforced polyetheretherketone, *SD* standard deviation, *D* dominant side, *ND* non-dominant side, *NSD* = Non significant difference (*p* > 0.05), *BMI* body mass index, *ASA* American Society of Anesthesiology Classification, *PVD* peripheral vascular disease, *NR* not reported, *NE* not extractable

Grouping the studies per anatomic area, generated four groups of studies:

(i) Fixation of proximal humerus fractures [19, 22, 25, 26].
(ii) Fixation of distal radius fractures [18, 21].
(iii) Fixation of distal femur fractures [23, 24].
(iv) Fixation of ankle fractures [20].

Analysing the patient characteristics, data for gender and age were available for almost all included studies (Table 2). Only for one study, data regarding age and gender were not extractable [26]. Eight studies that reported data for gender and age showed no significant difference between comparison groups. Four of the studies also reported data for patient comorbidities (BMI, smoking, diabetes, other medical problems) and comparison groups had no significant difference for the comorbidities reported (see Table 2) [19, 23–25].

## Methodological quality of studies and quality of evidence

There were two RCTs [18, 19] which were assessed for their methodological quality using the Cochrane Risk of Bias Tool [12]. The results of the assessment are shown in Table 3. One RCT had three key domains with unclear risk of bias (sequence generation, allocation concealment, blinding of the participants) [19]. The other study had adequate sequence generated, concealed allocation and blinding of participants without any other source of bias; hence, it can be classified as low risk of bias [18].

**Table 3:** Risk of bias of the RCTs with the Cochrane Risk of Bias Tool [10]

Lead author (year)	Sequence generation	Allocation concealment	Blinding of participants	Incomplete outcome data	Selective outcome reporting	Other source of bias	Total risk of bias
Ziegler (2019) [19]	Unclear	Unclear	Unclear	Yes	Yes	Yes	Unclear
Perugia (2017) [18]	Yes	Yes	Yes	Yes	Yes	Yes	Low

The Newcastle–Ottawa scale (NOS) was used to assess the methodological quality of the three prospective cohort studies included in the review [13]. The results are summarised in Table 4 below. All three studies were rated ‘good quality’ (for threshold see legend in Table 4), with two studies scoring the highest score of nine stars [20, 21]; and one study scoring seven stars [22].

**Table 4:** Risk of bias for prospective cohort studies using the Newcastle–Ottawa Scale (NOS) [11]

Lead Author (year)	Representativeness of cohort	Selection of non-exposed cohort	Ascertainment of exposure	Demonstration that outcome was not present at start of study	Comparability of cohorts	Assessment of outcome	Follow-up long enough for outcomes to occur	Adequacy of follow-up of cohorts	NOS score
Guzzini (2018) [21]	Truly representative*	Drawn from same community as the exposed cohort*	Secure record*	Yes*	Study controls for type of plate used* Study controls for gender, age*	Independent blind assessment*	Yes*	Complete follow up for all subject accounted for*	9 stars
Guzzini (2017) [20]	Truly representative*	Drawn from same community as the exposed cohort*	Secure record*	Yes*	Study controls for type of plate used* Study controls for gender, age*	Record linkage*	Yes*	Complete follow up for all subject accounted for*	9 stars
Katthagen (2017) [22]	Somewhat representative	Drawn from same community as the exposed cohort*	Secure record*	Yes*	Study controls for type of plate used* Study controls for gender, age*	Record linkage*	No	Subjects lost to follow up unlikely to introduce bias (< 20% lost)*	7 stars

A study can be awarded a maximum of 1 star for each question and a maximum of 2 stars for comparability of cohorts. The more stars a study was awarded, the lower was the risk of bias

Threshold for “good quality”: 3 or 4 stars in selection domain AND 1 or 2 stars in comparability domain AND 2 or 3 stars in outcome/exposure domain

The asterisks represent stars

The MINORS criteria were used to assess the methodological quality of the four retrospective cohort studies (Table 5) [14]. The lowest score was 18 out of 24 points for two of the studies [23, 26]. The highest score was 20 out of 24 points [25]. All studies clearly stated their aim, had adequate control and contemporary groups, and performed adequate statistical analysis. Three studies also included consecutive patients [24–26].

**Table 5:** Assessment of methodological quality of the retrospective cohort studies using MINORS criteria [12]

Criteria	Byun [23]	Mitchell [24]	Padolino [25]	Schliemann [26]
A clearly stated aim	2	2	2	2
Inclusion of consecutive patients	0	1	1	1
Prospective collection of data	0	1	1	2
Endpoints appropriate to the aim of study	2	2	1	2
Unbiased assessment of the study endpoint	2	2	2	2
Follow-up period appropriate to the aim of study	2	1	2	1
Loss to follow-up < 5%	2	2	2	1
Prospective calculation of the study size	0	0	1	0
Adequate control group	2	2	2	2
Contemporary group	2	2	2	2
Baseline equivalence of groups	2	2	2	1
Adequate statistical analysis	2	2	2	2
Total	18	19	20	18

The items are scored 0 (not reported), 1 (reported but inadequate) or 2 (reported and adequate)

Maximum possible score being 24 for comparative studies

*MINORS* Methodological Index for Non-randomized Studies

The GRADE approach was used to assess the overall quality of evidence in this review and the following ratings are reported [15]. The review included two RCTs but also seven non-randomised studies, so the starting rating of the study was ‘low quality’ evidence. The study had some inconsistency with methodological and clinical heterogeneity, having three different study designs, different anatomic areas for fixation, and differences in some population characteristics, even amongst the same study designs. However, there was no significant variability in the reported results. Overall, there were no concerns for indirectness, publication bias and imprecision. Based on this assessment, evidence is rated as ‘low quality’.

### Outcomes examined per anatomic area

#### Proximal humerus fractures

Four studies (patients *n* = 200) assessed outcomes of fixation of proximal humerus fractures and compared a CFR-PEEK humeral plate (Group 1) with a conventional Philos proximal humeral plate (Group 2/Control group) [19, 22, 25, 26]. One study was a RCT [19], one was a prospective cohort [22], and two were retrospective cohort studies [25, 26]. Two studies included 2-part, 3-part, and 4-part fractures (Neer classification [27]) in both groups [19, 22]. Interestingly, there were significantly more 3-part fractures fixed with the CFR-PEEK plate, whereas significantly more 4-part fractures were fixed with the conventional plate. The other two studies included only 3-part and 4-part fractures in both groups [25, 26]. The shorter radiological follow-up was 3 months [19]; the longest follow-up being 52.7 months (mean time) [25]. Outcomes are summarised in Table 6. There was no significant difference between comparison groups in clinical outcomes, ROM, and in the neck-shaft angle. Both groups in all studies had 100% union rate. With regards to complications there was no significant difference between two comparison groups. One study reported no complications in both groups [19]; one study reported the same complications (malunion, screw perforation, avascular necrosis, revision surgery) at almost the same low rate in both groups [25]. One study reported a lower rate of screw perforations and loss of fixations in the CFR-PEEK group as compared to the control group, although not significant [22]. Finally, one study reported again a lower rate of complications (malunions, AVN, revision surgery) in the CFR-PEEK group as compared to the control group, although not significant [26].

**Table 6:** Outcomes of studies examining proximal humerus fixation with CFR-PEEK plates

Lead author (year)	Comparison groups	Type of fractures (Neer) [13]	Clinical outcomes (Scores)	ROM	Radiographic outcomes	Union	Complications	Follow-up (months)/loss to follow-up
Ziegler (2019) [19]	Group 1 (*n* = 32) CFR-PEEK group Group 2 (*n* = 31) Control group	2-part Group 1: 6 Group 2: 5 NSD 3-part Group 1: 22 Group 2: 13 ***p* = 0.03** 4-part Group 1: 4 Group 2: 13 ***p* = 0.008**	DASH score Group 1: 27.5 ± 20.5 Group 2: 28.5 ± 17.9 Oxford score Group 1: 37.7 ± 8.8 Group 2: 38.6 ± 6.8 SST Group 1: 62.5 ± 22.3 Group 2: 65 ± 20.1	NR	Neck-shaft angle Group 1: 142.53° ± 6.45° Group 2: 138.81° ± 8.21°	Group 1 32/32–100% Group 2 31/31–100%	Malunion: 0 Screw perforation: 0 Loss of fixation: 0 Displacement: 0 AVN: 0 Implant failure: 0 Revision surgery: 0 Infection: 0 (both groups)	Clinical: 6 No loss Radiological: 3 Group 1: 28/31 Group 2: 23/31
Padolino (2018) [25]	Group 1 (*n* = 21): CFR-PEEK group Group 2 (*n* = 21): Control group	3-part: Group 1: 14 Group 2: 17 NSD 4-part: Group 1: 7 Group 2: 4 NSD	Constant score Group 1: 66.3 ± 20.5 Group 2: 63.3 ± 19.6 SST Group 1: 7 ± 2.25 Group 2: 7 ± 2.16	Active AE (NSD) Group 1: 142.8° Group 2: 127.6° Active LE (NSD) Group 1: 134.1° Group 2: 113.8° Ext. rotation (NSD) Group 1: 32.6° Group 2: 36.6° Int. rotation (NSD) Group 1: 2.8° Group 2: 2.4°		Group 1: 21/21–100% Group 2: 21/21–100%	Malunion Group 1: 2/21 (9.5%) Group 2: 2/21 (9.5%) Screw perforation: Group 1: 2/21 (9.5%) Group 2: 3/21 (14%) AVN Group 1: 1/21 (4.8%) Group 2: 1/21 (4.8%) Revision surgery- Group 1: 1/21 (4.8%) Group 2: 1/21 (4.8%)	Clinical/ Radiological Group 1: Mean: 30.7 Range: 24–54 Group 2: Mean: 52.7 Range: 29–77 No loss
Katthagen (2017) [22]	Group 1 (*n* = 21): CFR-PEEK group Group 2 (*n* = 21): Control group	2-part: Group 1: 2 Group 2: 2 NSD 3-part: Group 1: 9 Group 2: 12 NSD 4-part: Group 1: 10 Group 2: 7 NSD	Constant score Group 1: 73.8 ± 15.4 Group 2: 69.4 ± 18.5 *p* = 0.43 SSV: Group 1: 0.74 VAS pain: Group 1: 0.1 ± 0.4	Abduction Group 1: 124.3° ± 42° Group 2: NR	Neck-shaft angle: Group 1: 129.6° ± 8.7° Group 2: NR	Group 1: 17/17–100% Group 2: NR	Malunion: 0 (both groups) Screw perforation: (NSD) Group 1: 0/17 Group 2: 5/19 (26%) Loss of fixation: (NSD) Group 1: 0/17 Group 2: 3/19 (16%) Displacement Group 1: 0 Group 2: NR AVN: Group 1: 0 Group 2: NR Implant failure Group 1: 0 Group 2: NR Revision surgery: (NSD) Group 1: 4/21 (19%) Group 2: 5/21 (24%)	Clinical: 12 Group 1: 20/21 Group 2: 19/21 Radiological: Mean: 3.2 Range: 1.5–5 Group 1: 17/21
Schliemann (2015) [26]	Group 1 (*n* = 23): CFR-PEEK group Group 2 (*n* = 30) Control group (historical)	3-part 4-part	Constant score (NSD) Age/Gender adjusted Group 1: 71.3 (44–97) Group 2: 59.2 (28–86) DASH score (NSD) Group 1: 27.5 (7–48) Group 2: 28.5 (10.6–46.4) Oxford score (NSD) Group 1: 27.4 (8–45) Group 2: 21.6 (9–43) SST Group 1: 59 Group 2: 48	Abduction Group 1: 145° (120°–150°) Group 2: NR Active AE Group 1: 170° (150°–180°) Group 2: NR	Neck-shaft angle: Group 1: 139° (129°–146°)	Group 1: 100% Group 2: 100%	Malunion: (NSD) Group 1: 4/23 (14%) Group 2: 7/30 (23%) Screw perforation: 0 (both groups) Loss of fixation: 0 (both groups) Displacement 0 (both groups) AVN: (NSD) Group 1: 1/23 (4%) Group 2: 3/30 (10%) Implant failure 0 for both groups Revision surgery: (NSD) Group 1: 7/23 (30%) Group 2: 8/30 (27%)	Clinical: 24 Group 1: 23/29 Radiological: 6 Group 1: 29/29

*CFR-PEEK* carbon fibre-reinforced polyetheretherketone, *ROM* range of motion, *DASH* disabilities of the arm, shoulder and hand, *VAS* Visual Analogue Scale, *SSV* Simple Shoulder Value, *SST* Simple Shoulder Test, *NR* not reported, *NSD* no significant difference, *AE* anterior elevation, *AVN* avascular necrosis

Statistically significant values are in bold

#### Distal radius fractures

Two studies (patients *n* = 74) assessed outcomes of fixation of distal radius fractures with a CFR-PEEK plate [18, 21]. One study (RCT) compared the CFR-PEEK volar locking plate (Group 1) with a conventional titanium volar locking plate (Group 2/Control group) [18]. The other study (prospective cohort) compared the operated side using the CFR-PEEK volar locking plate (Group 1) with the uninjured contralateral side (Group 2/Control group) [21]. The mean time for follow-up was 15.7 months for both studies. Outcomes are summarised in Table 7. One study reported that no patients in both groups had a significant difference of grip strength, hand grip and key pinch as compared to the contralateral side [18]. The DASH score, the time to return to activities of daily living (ADL), and the Visual Analogue Scale (VAS) for pain had no significant difference between comparison groups. There was no significant difference between groups in wrist ROM as well. All radiographic values (radial height, radial inclination, volar tilt, ulnar variance, articular step-off) showed no significant difference between groups. There were also no complications reported for both groups. The second study reported no significant difference for grip strength and hand grip as compared to the contralateral uninjured side [21]. ROM also had no significant difference as compared to the contralateral side. This study reported on union rates and all fractures treated with the CFR-PEEK volar locking plate were united (100%). There were no complications reported in both studies.

**Table 7:** Outcomes of studies examining distal radius fixation with CFR-PEEK plates

Lead author (year)	Comparison groups	Type of fractures (ΑΟ classification)	Clinical outcomes	ROM	Radiographic outcomes	Union	Complications	Follow-up (months)/loss to follow-up
Guzzini (2018) [21]	Group 1 (*n* = 22): CFR-PEEK group Group 2 (*n* = 22): Control group	NR	QuickDASH Group 1: 9.3 (2.5–15.9) Hand Grip Group 1: 92.3% Mean: 19.5 kg Group 2: NSD Key pinch Group 1: 90.4% Mean: 8.1 kg Group 2: NSD Return to ADL Group 1: mean 4.2 weeks VAS Group 1: 2.3 (0–3.5)	Extension Group 1: 65° (54°–76°) Group 2: NSD Flexion Group 1: 70° (72°–80°) Group 2: NSD Supination Group 1: 87° (82–90) Group 2: NSD Pronation Group 1: mean 80° Group 2: NSD	Normal radial height Group 1: 70.6% (6.8–7.3 mm) Normal radial inclination Group 1: 78.5% (15–27.5°) Normal volar tilt Group 1: 93.2% (3–187°) Ulnar variance Group 1: 89.5% (0.7–4.1 mm) Articular step-off Group 1: 18%	Group 1: 22/22–100%	None	Clinical/radiological Mean: 15.7 Range: 12–19
Perugia (2017) [18]	Group 1 (*n* = 15): CFR-PEEK group Group 2 (*n* = 15): Control group	B1 Group 1: 2 Group 2: 1 NSD B2 Group 1: 1 Group 2: 0 NSD B3 Group 1: 3 Group 2: 1 NSD C1 Group 1: 5 Group 2: 4 NSD C2 Group 1: 1 Group 2: 3 NSD C3 Group 1: 3 Group 2: 6 NSD	DASH (NSD) Group 1: 15.3 (2.5–58.9) Group 2: 12.2 (10.6–54.8) Hand grip (NSD) Group 1: 92.3% Mean: 19.5 kg Group 2: 94.4% Mean: 22.4 kg Key pinch (NSD) Group 1: 90.4% Mean: 8.1 kg Group 2: 90.7% Mean: 8.4 kg Return to ADL (NSD) Group 1: mean 4.2 weeks Group 2: mean 3.8 weeks VAS (NSD) Group 1: mean 3.6 (0–7) Group 2: mean 2.9 (0–6)	Extension (NSD) Group 1: 64° (44°–76°) Group 2: 61° (42°–75°) Flexion (NSD) Group 1: 78° (59°–80°) Group 2: 80° (62°–80°) Supination (NSD) Group 1: 87° (72°–90°) Group 2: 88° (70°–90°) Pronation (NSD) Group 1: 80° Group 2: 77°	Normal radial height Group 1: 66.6% (6.8–17.3 mm) Group 2: 70% (6.3–18.2 mm) Normal radial inclination Group 1: 75% (15–27.5°) Group 2: 73% (14–29°) Normal volar tilt Group 1: 90.2% (3–187°) Group 2: 91.3% (5–185°) Ulnar variance Group 1: 86.3% (0.7–4.1 mm) Group 2:85.8% (0.5–4.8 mm) Articular step-off Group 1: 35.3% Group 2: 37% NSD for all values	Not reported	None	Clinical/ Radiological Group 1: Mean: 15.7 Range: 12–19 No loss to f/u Group 2: Mean: 16.1 Range: 13–21 No loss

*CFR-PEEK* carbon fibre-reinforced polyetheretherketone, *DASH* disabilities of the arm, shoulder and hand, *ADL* activities of daily living, *VAS* Visual Analogue Scale, *NR* not reported, *NSD* not significant difference

#### Distal femur fractures

Two studies (patients *n* = 53) assessed outcomes of fixation of distal femur fractures and compared a CFR-PEEK distal femur locking plate (Group 1) with a conventional stainless steel variable angle distal femur LCP plate (Group 2/Control group) (Table 8) [23, 24]. Both studies were retrospective cohort studies. There was no significant difference in the type/severity of fractures between groups (OTA Compendium classification). One study had a follow-up of 6 months [23], and the other study had a longer follow-up with a mean time of 12.25 months. Outcomes are summarised in Table 8. Regarding union of the fractures, one study used the modified RUST (mRUST) score with no significant difference between the two plates [23]. All the fractures were united in the CFR peek group, whereas the control group had three cases (14%) of non-union. The second study reported on the mean time to radiographic union which showed no significant difference between two plates [24]. The CFR-PEEK plate group had only one case of non-union, and the conventional stainless steel plate group had four cases of non-union, with the difference being not significant. Regarding complications, both studies showed no significant difference between groups in the few complications reported (hardware failure, reoperation, change in alignment).

**Table 8:** Outcomes of studies examining distal femur fixation with CFR-PEEK plates

Lead author (year)	Comparison groups	Type of fractures (OTA compendium classification)^x^	Union	Non-union	Mean time to FWB	Complications	Follow-up (months)/loss to follow-up
Byun (2020) [23]	Group 1 (*n* = 10): CFR-PEEK group Group 2 (*n* = 21): Control group	Type C Group 1: 7 Group 2: 14 (*p* = 0.972) Periprosthetic Group 1:2 Group 2: 5 (*p* > 0.05) Open Group 1: 4 Group 2: 9 (*p* = 0.597) Closed Group 1: 6 Group 2: 12 (*p* > 0.05)	mRUST score Group 1: 11.4 ± 2.6 (7.7–16) Group 2: 10.5 ± 2.5 (6.0–15.7) (*p* = 0.374)	Group 1: 0/10 Group 2: 3/21 (14%)	NR	Hardware failure 0 in both groups Reoperation Group 1: 0/10 Group 2: 3/21 Change in alignment Group 1: 1/10 (10%) Group 2: 1/21 (4.8%) (*p* = 0.548)	Clinical/radiological 6 months No loss
Mitchell (2018) [24]	Group 1 (*n* = 11): CFR-PEEK group Group 2 (*n* = 11): Control group	Type C Group 1: 4 Group 2: 5 (*p* = 0.68)	mRUST score N/R Mean time to radiographic union Group 1: 18.8 weeks Group 2: 12.4 weeks (*p* = 0.14)	Group 1: 1/11 (9%) Group 2: 4/11 (36%) (*p* = 0.12)	Group 1: 9.8 Group 2: 11.7 (*p* = 0.12)	Hardware failure Group 1: 0/11 Group 2: 2/11 (*p* = 0.14) Reoperation Group 1: 1/11 (9%) Group 2: 4/11 (36%) (*p* = 0.08) Change in alignment NR	Clinical/ Radiological Group 1: Mean: 12.25 Range: 2.5–15 No loss Group 2: Mean: 11.5 Range: 2.5–30.5 No loss (*p* = 0.82)

*CFR-PEEK* carbon fibre-reinforced polyetheretherketone, *OTA* Orthopaedic Trauma Association, *FWB* full weight bearing, *ROM* range of motion, *NR* not reported, *mRUST* modified radiograph union score

#### Ankle fractures

One study (*n* = 87) assessed the outcomes of fixation of ankle fractures and compared a CFR-PEEK ankle plate (Group 1) with a conventional stainless steel ankle plate (Group 2/Control group) [20]. The mean time to follow-up was 14 months. Regarding clinical outcomes (Table 9), there was no significant difference between the comparison groups for all outcomes/scores reported (Olerud-Molander Ankle score, Ankle-Hind foot scale, VAS). All reported radiographic values (Talocrural angle, restoration of joint line) showed no significant difference between two groups. There was no significant difference reported between the two plates for the time to union, but the authors did not give any numbers. Three patients (6.5%) in the CFR-PEEK group and four patients (9.8%) in the control group required removal of metalwork with the difference being not significant.

**Table 9:** Outcomes of studies examining ankle fracture fixation with CFR-PEEK plates

Lead author (year)	Comparison groups	Clinical outcomes	Radiographic outcomes	Time to union	Removal of metalwork	Follow-up (months)/Loss to follow-up
Guzzini (2017) [20]	Group 1 (*n* = 46): CFR-PEEK group Group 2 (*n* = 41): Control group	OMAS Group 1: 91.1 ± 4.16 Range: 86–95.26 Group 2: 88.7 ± 4.7 Range 84–93.4 NSD AOFAS Group 1: 92.1 ± 4.16 Range 87.94–96.26 Group 2: 90.1 ± 4.7 Range 85.4–94.7 NSD VAS Group 1: 1.4 ± 1.1 Range 0.3–2.5 Group 2: 1.5 ± 0.7 Range 0.8–2.2 NSD	Talocrural angle Group 1: 9.3 ± 0.9° Range 8.4–10.2° Group 2: 10.4 ± 0.8° Range 9.6–11.2° NSD Restoration of joint line Group 1: 45/46 Group 2: 39/41 NSD	NSD between 2 groups	Group 1: 3/46 (6.5%) Group 2: 4/41 (9.8%) NSD	Clinical/radiological Mean: 14 ± 2 Range: 6–24 No loss

*CFR-PEEK* carbon fibre-reinforced polyetheretherketone, *OMAS* Olerud-Molander Ankle score, *AOFAS* Ankle-Hindfoot scale, *VAS* Visual Analogue Scale, *NSD* not significant difference

## Discussion

Although CFR-PEEK implants have been around for years and have several advantages [4, 8, 18–20, 23, 25, 26], they are slowly regaining popularity and have been used in a variety of orthopedic applications including trauma, infection, and tumors [5]. This is the first systematic review providing evidence regarding the use of CFR-PEEK plates in extremity trauma. Overall results indicate very high union rates similar to conventional plates when used for fixation of either upper or lower limb fractures with similarly good clinical outcomes/scores. The rate of complications is low and comparable to that reported in the literature for their conventional counterparts.

For this review only Level I–III evidence studies, both randomised and non-randomised, were included. However, there were enough retrospective case series studies (Level IV) that were excluded from the analysis. During screening of the available evidence, ten relevant retrospective case series studies were identified [4, 6, 8, 28–34]. Acknowledging the limitations of such study designs, it is worth to summarise and note their findings on the use of CFR-PEEK plates in fracture fixation. The characteristics and the findings of these case series studies are summarised in Table 10.

**Table 10:** Characteristics and findings of excluded retrospective case series studies

Lead Author (year)	Implants/company/country	Sample size (*n*)	Gender	Age (years)	Type of fractures	Follow-up (months)/loss to follow-up	Union/time to union	Outcomes/complications
Proximal humerus								
Rotini (2015) [4]	Diphos H CFR-PEEK plate/Lima Corporate/Italy	160	41 M:119F	Mean:64 Range: 23–84	Neer (13) 2-part: 55 3-part: 76 4-part: 29	Minimum f/u: 24 Lost to f/u: 12%	Union: 158/160 (99%) Time: NR	Outcomes: Mean Constant Score: 76 Mean DASH Score: 28 Abduction: 129° ± 25° Active AE: 137° ± 28° Ext. rotation: 48° ± 19° Int. rotation: 56° ± 26° Complications: Plate breakage (intraoperatively): 3/160 (2%) (1st generation plates) Screw perforation: 8/160 (5%) Loss of fixation: 5/160 (3%) Fragment displacement: 2/160 (1.3%) AVN: 13/160 (8%) Implant failure: 5/160 (3%) Revision surgery: 41/160 (25.6%)
Distal radius								
Tarallo (2020) [34]	Volar fixed angle plate DiPHOS-RM/Lima Corporate/Italy	110	33M:77F	Mean:56.8 Range: 23–84	AO: A3 (14). B3 (33), C1 (18), C2 (30), C3 (15)	Mean: 48 Range: 14–81 Lost to f/u: 9%	NR	Outcomes: Not reported (only analyzed adverse events) Complications: Intraop plate ruptures: 4/110 (3.6%) (revised with new PEEK plate) FPL rupture: 1 Revision surgery: 4/110 (3.6%) 1 for post-op plate rupture 2 for extensor irritation 1 for infection
Allemann (2019) [29]	2.7 mm CF/PEEK plate/Inc. Icotec, Altstätten/Switzerland	10	6M:4F	Mean: 53.3 ± 16.6	AO All type B fractures	Minimum f/u: 12 Loss to f/u: NR	Union: 10/10 (100%) Time: NR	Outcomes: Wrist ROM: significant increase Return to ADLs without limitations: 10/10 (100%) Complications: Intraop breakage of screws: 2 (20%)
Di Maggio (2017) [8]	Piccolo Composite™ CFR-PEEK radiolucent volar plate/Unimedical Biomedical Technologies/Italy	64	38M:26F	Mean: 56.8 Range: 23–84	AO: B1 (6), B2 (13), B3 (15), C1 (10), C2 (7), C3 (10)	Minimum f/u: 12 Lost to f/u: 9.8%	Union: 64/64 (100%) Time to union: 6 weeks	Outcomes: Modified Mayo wrist Score: 90.54 (range 75–100; 95% CI: 88.4–92.6) Return to ADLs without limitations: 64/64 (100%) Complications: Plate removal: 1/64 (1.6%) (aseptic loosening of screw)
Tarallo (2014) [6]	Volar fixed angle plate DiPHOS-RM/ Lima Corporate/Italy	40	16M:24F	Mean: 65 Range: 26–82	AO: B1 (2), B2 (6), C1 (21), C2 (9), C3 (2)	Minimum f/u: 12 Loss to f/u: NR	Union: 40/40 (100%) Time: NR	Outcomes: Mean DASH: 6 (3–16) Grip strength: 92% of contralateral Return to ADLs without limitations: 40/40 (100%) Extension: 55°(40°–65°) Flexion: 65°(45°–80°) Supination: 75°(65°–90°) Pronation: 79° (60°–90°) Complications: Plate removal: 1 (2.5%) (flexor tenosynovitis—technical error)
Distal femur								
Baker (2004) [30]	Distal femur carbon plate OrthoDesign/OrthoDynamics, UK	12	NR	Mean: 78 Range: 57–94	NR	NR	11/12 (85%) Time to union: 4 months (3–6)	Outcomes: Return to pre-injury level of mobility: 11/12 (92%) Complications: Νon-union: 1/12 (8%) (revised with long stem prosthesis)
Al-Shawi (2002) [28]	NR/UK	5	5F	Mean: 74.8 Range: 69–83	NR	Mean: 30 Range: 18–42	Union: 5/5 (100%) Time to union: 5 weeks–5 months (mean)	Outcomes: No residual pain Complications: Malunion: 1 (clinically not significant)
Pemberton (1994) [32]	Distal femur carbon plate OrthoDesign/OrthoDynamics/ UK	19	19F	Mean: 80 Range: 66–92	AO A2 (9), A3 (8), C2 (2)	Minimum f/u: 12 Loss to f/u: NR	17/19 (89%) Time to union 2–5 months	Outcomes: Return to pre-injury level of mobility and independence: 17/17 Complications: Shortening (1–1.5 cm): 2/19 (10.5%) Union in 10° varus: 1/19 (5%) < 90° flexion: 2/19 (10.5%)
Ankle								
Pinter (2018) [33]	Piccolo CompositeTM Distal Fibula Plate/ Carbofix Orthopedic Ltd/Israel	30	12M:18F	Mean: 46.8 Range: 18–79	Weber B (27) Weber C (3)	Mean: 20 Range: 12–27 Lost to f/u: 20%	23/24 (96%)	Outcomes: No outcome scores reported Complications: Failure of syndesmosis fixation: 1/30 (3%) Infection: 1/30 (3%)
Caforio (2014) [31]	Piccolo CompositeTM Distal Fibula Plate/Carbofix Orthopedic Ltd/Israel	27	14M:13F	Mean: 57.3 Range: 19–78	Monomalleolar: 4 Bimalleolar: 12 Trimalleolar: 11	Minimum f/u: 3 Loss to f/u: NR	NR	Outcomes: Full recovery of ROM: 26/27 (96%) No pain (at 2 months): 26/27 (96%) Complications: Plate removal: 2 (7%) Skin discoloration: 1 (3.7%) Reduced ROM: 1 (3.7%)

*UK* United Kingdom, *M* males, *F* females, *f/u* follow-up, *NR* not reported, *AE* anterior elevation, *int* internal, *ext* external, *AVN* avascular necrosis, *ROM* range of motion, *ADLs* activities of daily living, *FPL* flexor pollicis longus

### Proximal humerus fractures

The results reported herein, indicate that there is no significant difference in clinical outcomes, ROM and neck-shaft angle, and there is a 100% union rate [4, 19, 22, 25, 26] with a similar complication rate. In a retrospective study, a high union rate was also observed (Table 10) [8]. However, a higher incidence of complications was noted (plate breakage and revision surgery) but these findings can be attributed to the first generation of CFR-PEEK plates used. The proximal humerus has several particularities when conventional implants are used: The high-rigidity of titanium implants may lead to 8–12% secondary screw perforation, and subsequent loss of reduction with up to 20% revision rates, especially when the bone is osteoporotic [35, 36]. In addition, primary screw perforation has been reported in up to 8% of cases due to the intraoperative difficulty of visualizing the posterior humeral head as it is obscured by the radio-opaque hardware [35–37]. In theory therefore, the use of a CFR-PEEK plate is advantageous. Indeed, two studies reported a trend with lower complications of CFR-PEEK plates, including screw perforation and loss of fixation [22], and malunions, AVN and revision surgeries [26], however this did not reach statistical significance. It can be concluded that the use of CFR-PEEK plates in the proximal humerus is justified with equivalent results and potentially lower complication rates, but further larger-scale studies are needed to confirm or dismiss those trends.

### Distal radius fractures

The two studies included in the review, showed that the results of the CFR-PEEK plates are comparable to either the conventional implant (*n* = 44) [21] or to the contralateral side (*n* = 30) [18] respectively and reported no complications at a minimum of 12-month follow-up. However, four retrospective case series not included in this systematic review (*n* = 224) reported on distal radius fracture fixation with a volar CFR-PEEK plate with a minimum of 12-month of follow-up (Table 10) [6, 8, 29, 34]. One study had only ten patients [29]. The remaining three studies (*n* = 195) which reported on union of the fractures, showed 100% of union rate [6, 8, 29]. The same studies reported that all patients returned to activities of daily living (ADLs) with no limitations and had good clinical outcomes/scores (Mayo wrist score or DASH score) [6, 8, 29]. All studies reported a very small number of complications. Specifically relating to the CFR-PEEK plate, one study reported a rate of 3.6% of intraoperative plate rupture that was replaced with a new one without further complications as well as a rate of 0.9% of post-operative plate rupture requiring revision [34]. Of note, plate ruptures occurred when there was overtightening of a 3.5-mm cortical screw in the diaphyseal elliptical hole or a 2.7-mm locking screw on the radial side where the plate is weaker as there is a K-wire hole there. The authors thus recommended careful technique to avoid overtightening the screws [34]. This is backed up by a biomechanical study where the CFR-PEEK plate was found to have lower tolerance to plastic deformation induced by compressive forces compared to titanium or stainless-steel distal radius plates [38].

In the distal radius area in particular, the radiolucent nature of the CFR-PEEK plate is helpful for adequate intraoperative anatomical fracture reduction, especially when multiple fragments are involved, and in addition, the lack of MRI artefact would be useful in better assessing soft-tissue pathology that is close to the plate [8, 34] and frequently associated with these fractures (e.g., TFC tears) [39]. It can be concluded that overall, the use of CFR-PEEK plates in distal radius fractures is supported in the literature with excellent outcomes and similar complications to conventional plating, with the need of further studies to confirm it. Although plate rupture is a rare phenomenon, knowledge of the biomechanical properties described above and careful technique is of paramount importance to avoid plate rupture.

### Distal femur fractures

Fractures in this region are challenging to treat with non-union rates using a lateral locking plate of up to 20% [40], despite different attempts to modify hardware and technique to achieve some micro-motion at the fracture site [24]. Therefore, CFR-PEEK plates with a modulus of elasticity closer to bone may offer this advantage and optimize union rates. In the two studies analyzed herein (Table 8), there was an incidence of 14% non-union for the control group vs 0% in the CFR-PEEK group in one [23], and 36% versus 9% respectively in the other study, as well as decreased time to union, although these did not reach statistical significance [24]. Similarly, there were trends with higher implant failure and rates of reoperation in the control group versus the CFR-PEEK group, but again non-significant [24]. Three retrospective case series (*n* = 36) (Table 10) reported on distal femur fracture fixation with a carbon fiber plate [28, 30, 32]; with one study having only five female patients [28]. Union rates ranged from 85% in one study [30], to 100% in another study [28]. Time to union ranged from 5 weeks to 5 months with a low number of complications reported. Implant failure is extremely rare with only two cases reported in the literature [7, 30]. It may be concluded that the use of CFR-PEEK plates for distal femoral fractures is justified having a low complication rate and is particularly promising with regards to lowering non-union rates. However, the trends found in the literature should be confirmed by higher quality studies.

### Ankle fractures

Ankle fractures are sometimes challenging to treat and in particular when dealing with complex trimalleolar fractures, a radiolucent CFR-PEEK plate has the advantage of adequate visualization of the posterior malleolus after fixation of the fibula to ensure anatomical reduction of the joint surface [31]. In the study reported herein (*n* = 87) [20], a CFR-PEEK plate fixation of the lateral malleolus showed equivalent clinical and radiographic results to the control group at 6 months minimum follow-up. In addition, two retrospective case series (*n* = 57) (Table 10) evaluated ankle fracture fixation with a distal fibula carbon fiber plate [31, 33]. Both studies reported very low number of complications not specific to the implant. One study had excellent outcomes (full recovery of ROM and no pain) with a minimum follow-up of 3 months [31]. The other study reported an excellent rate of union (96%) [33]. It may be concluded that, with their added advantages, the use of CFR-PEEK fibular plates in the ankle is recommended, with outcomes and complications similar to the conventional implants.

This study has its own limitations. The overall quality of evidence in this review is limited to ‘low quality’ by the low quality of the included studies and data. There were three different study designs with an overlap of prospective and retrospective data with heterogeneous studies examining different anatomical areas (proximal humerus, distal radius distal femur, ankle) and with small numbers, making a meta-analysis impossible. These limitations are expected since the use of CFR-PEEK plates in extremity trauma has only recently received more attention. Nonetheless, we followed all the principles governing systematic review design and evaluation the heterogeneity and risk of bias components.

There are several strengths of this study. Firstly, only level III and above comparative studies were included, and the majority of them were level I or II. It is comprehensive as the ten retrospective case series that were excluded from the analysis, are presented in the discussion section by comparing and contrasting their findings with the included studies to ensure an adequate overview of the subject. Finally, it is the only study focusing exclusively on plates for extremity fracture fixation, in contrast to previous more generic publications [2, 3, 5], and will serve as a complete, up-to-date reference for their current status.

## Conclusions

Our study compared the outcomes and complications of fracture fixation with CFR-PEEK plates versus conventional plates in the proximal humerus, distal radius, distal femur, and ankle. CFR-PEEK plates have major advantages over conventional plates, include a modulus of elasticity comparable to bone, radiolucency, decreased artefact on CT and MRI, higher biocompatibility with absence of allergies and inflammatory reactions and no cold-welding. Our findings show that CFR-PEEK plates have very high union rates in extremity fracture fixation similar to conventional plates with comparable good clinical outcomes and a very low and comparable rate of complications. Future larger scale prospective studies could provide further robust evidence of their benefits. Considering their advantages, which are reflected in favourable not yet statistically significant trends across different anatomical regions, CFR-PEEK plates seem to be a valid alternative to conventional plating.

## Supplementary Information

Below is the link to the electronic supplementary material.

Supplementary file1 (DOCX 32 KB)

## Data Availability

All associated extracted data are stored and available upon request.
